# Suppressive Effects of Turmeric Extract on Muscle Atrophy in Dexamethasone-Treated Mice and Myotubes

**DOI:** 10.3390/nu14193979

**Published:** 2022-09-25

**Authors:** Kyohei Furukawa, Marika Kousaka, Huijuan Jia, Hisanori Kato

**Affiliations:** Health Nutrition, Department of Applied Biological Chemistry, Graduate School of Agricultural and Life Sciences, The University of Tokyo, 1-1-1 Yayoi, Bunkyo-ku, Tokyo 113-8657, Japan

**Keywords:** sarcopenia, Fbxo32, MuRF1, C2C12, glucocorticoid, turmeric, curcuma longa

## Abstract

Sarcopenia is the decline in skeletal muscle mass, strength, and functions, which decreases the quality of life in elderly people. This study investigated the suppressive effect of turmeric (Curcuma longa) extract (TE) on muscle atrophy in dexamethasone (DEX)-treated mice and C2C12 myotubes. DEX treatment significantly decreased the muscle weight and significantly increased Fbxo32 and Murf1 expression in mice, and these changes were suppressed by the supplementation of an AIN-93 based diet with 2% TE. A similar pattern was observed in FBXO32 and MuRF1 protein expression. In C2C12 myotubes, DEX treatment significantly increased FBXO32 and MuRF1 gene and protein expression, and these increases were significantly suppressed by TE supplementation at a concentration of 200 µg/mL. Furthermore, one of the five TE fractions, which were separated by high-performance liquid chromatography had a similar effect with TE supplementation. The present study proposes the suppressive effect of turmeric on sarcopenia.

## 1. Introduction

The aging population is increasing rapidly, and the global population aged over 60 is expected to increase by 22% in 2050. Aging affects cellular metabolism, physiology, and function, and these changes result in increased disease morbidity and mortality and decreased quality of life (QoL). Skeletal muscle has a vital role not only in locomotion but also in energy metabolism and protein reservoir. Aging declines muscle mass, strength, and performance [1], and these physiological changes are known as sarcopenia [2,3], which decreases QoL. Therefore, the prevention and improvement of sarcopenia are vital to achieve “healthy aging” [4].

The mass of skeletal muscle varies according to physiological and pathological conditions. In adulthood, the muscle mass is reflected by protein turnover, which is the balance between protein synthesis and degradation. Thus, muscle atrophy results from an imbalance in protein metabolism. Several studies have shown that F-Box Protein 32 (FBXO32) and muscle RING-finger protein-1 (MuRF1), which are muscle-specific ubiquitin ligases, are upregulated in muscle atrophy [5,6,7,8]. The knockout mice of these genes were resistant to muscle atrophy [9]. Furthermore, the expression of FBXO32 and MuRF1 is mediated by several factors, such as oxidative stress, insulin-like growth factor-1 (IGF-1), glucocorticoid, and inflammatory cytokines, through the Forkhead box O (FoxO) signaling pathway [5,10].

Turmeric (Curcuma longa) is used not only as a yellow spice common in Indian cuisine but also as traditional medicine against gastrointestinal disorders and arthritic pain. Indeed, several nutritional studies indicated that turmeric, particularly its curcuminoid component curcumin, has anti-inflammatory, antioxidative stress, and antidiabetic properties [11,12,13]. Hence, curcumin is an important component of turmeric against several diseases and physiological changes. Furthermore, bisacurone, a sesquiterpenes in turmeric, suppresses hepatic lipid accumulation and has a beneficial role in the treatment of inflammatory diseases [14,15]. Thus, turmeric and its components have an important role in mediating health and physiological changes. However, few studies have investigated their role in muscle atrophy or sarcopenia, except for one study that showed curcumin ameliorated muscle atrophy in type I diabetes mice [16]. The present study investigated the effect of turmeric extract (TE) on muscle atrophy in dexamethasone (DEX)-treated mice and C2C12 myotube.

## 2. Materials and Methods

### 2.1. Preparation of TE and TE Fraction

TE was extracted via water extraction from the rhizomes of C. longa which is originally from China, and dried under reduced pressure. The analysis using high performance liquid chromatography (HPLC) showed that the extract contained more than 0.15% of bisacurone and less than 0.1% of curcuminoid. For the preparation of TE fraction, TE was mixed with 90% methanol, and the ethyl acetate fraction was collected. The fractions were further separated into five fractions via HPLC under 50% and 100% of methanol elution. We obtained 11.0, 6.8, 6.8, 5.0, and 14.7 g of fraction 1 (Fr.1), Fr.2, Fr.3, Fr.4, and Fr.5, respectively, from 2 kg of TE powder.

### 2.2. Animal Experiments

Fifteen-week-old male C57BL/6J mice were fed an AIN93-based diet or 2% TE supplemented AIN93-based diet. Five days later, 0.9% of saline or 5 mg/kg body weight of DEX was intraperitoneally administered every day for a week. The mice were individually housed in cages in a temperature-controlled (22 ± 1 °C), 12 h light–dark cycle environment and provided ad libitum access to drinking water and diet. After the experimental period, biopsies from five types of skeletal muscle (gastrocnemius, plantaris, tibialis anterior, extensor digitorum longus, and soleus) were collected under deep anesthesia with isoflurane and stored at −80 °C until further analysis. In this study, total muscle weight was calculated as the sum of the weights of the muscles mentioned above. The diet composition is shown in Table S1.

### 2.3. Cell Caltivation

C2C12 cells were incubated with Dulbecco’s modified Eagle medium containing 10% fetal bovine serum and 1% penicillin-streptomycin under 37 °C and 5% CO2 conditions. The cells were seeded in 6-well collagen I-coated plates for Western blotting analysis and in 24-well plates for real-time reverse transcription polymerase chain reaction (RT-PCR) and lactate dehydrogenase (LDH) assay. Upon reaching 90% confluence, the cells were incubated with 4% horse serum-supplemented medium for cell differentiation. After 6 d, the cells were incubated with serum-free medium for 12 h and treated with or without 100 nM DEX, 200 µg/mL of TE, and five TE fractions for 12 h.

### 2.4. Isolation of Total RNA and Real-Time RT PCR Analysis

The skeletal muscle sample was homogenized with 600 µL of lysis buffer. Total RNA was then isolated from half of the lysate (300 µL) using RNeasy Tissue Mini Kit (Qiagen, Hilden, Germany) following the manufacturer’s instructions.

Meanwhile, C2C12 cells were washed with phosphate-buffered saline and lysed with 300 µL of lysis buffer including 1% of 2-mercaptoethanol. Total RNA was then isolated from the cells using RNeasy Fibrous Tissue Mini Kit (Qiagen) following the manufacturer’s instructions.

Total RNA concentration was measured using NanoDrop ND-1000 (Thermo Fisher Scientific, Waltham, MA, USA). Total RNA (500 ng) was reverse transcribed to cDNA using PrimeScript™ RT Master Mix (Takara Bio Inc., Shiga, Japan) according to the manufacturer’s instructions. The synthesized cDNA was mixed with appropriate primers and SYBR^®^ Premix Ex Taq™ (Takara Bio Inc., Shiga, Japan), and real time RT-PCR was performed on a Thermal Cycler Dice Real Time System TP800 (Takara Bio Inc.). The primer sequences are shown in Table S2. The data were normalized to peptidyprolyl isomerase A (Ppia) mRNA levels. The results are expressed as fold-change values compared with the control.

### 2.5. DNA Microarray Analysis

Total RNA from the tibialis anterior muscle of three mice, which had a body and muscle weight close to the average value, was used for DNA microarray analysis according to previous studies [17,18]. RNA samples (250 ng) were reverse transcribed into single-stranded cDNA using GeneChip^®^ 3′IVT Express Kit (Thermo Fisher Scientific). Synthesis of double-stranded cDNA and complementary RNA (cRNA) was performed following the manufacturer’s instructions. The synthesized cRNA was hybridized onto the array chip at 45 °C for 16 h and stained with streptavidin-phycoerythrin using GeneChip™ Fluidics Station 450 (Applied Biosystems™, Foster City, CA, USA). Images were obtained using GeneChip™ Scanner 3000 (Affymetrix, Santa Clara, CA, USA).

The obtained CEL file data was normalized using Robust Multi-array Average algorithm implemented in R software (https://www.R-project.org/, accessed 1 October 2021, version 3.6.2). Comparison between the two groups was performed via Rank Product analysis. Gene probes with a signal intensity of less than 50 were removed. The cut-off conditions were False Discovery Rate <0.05 and |log ratio| ≥ 0.6. The gene sets satisfying the cut-off conditions were further analyzed using Ingenuity Pathway Analysis software (http://www.ingenuity.com/, accessed 1 October 2021).

### 2.6. Western Blotting Analysis 

For assays using animals, we selected four mice from each group that had a body and muscle weight close to the average value. The muscle and cells were lysed with radioimmunoprecipitation assay buffer containing 25 mM of Tris-HCl (pH 7.6), 150 mM of NaCl, 1% of NP-40, 0.1% of sodium dodecyl sulfate (SDS), 1% of sodium deoxycholate, 1 KIU of aprotinin, 10 mM of Na3VO4, and 0.2% of Protease Inhibitor Cocktail (NACALAI TESQUE Inc., Kyoto, Japan). The lysate was rotated under 4 °C for 1 h and sonicated three times at 10 s each. The sample was centrifuged at 16,000× *g*, 4 °C for 30 min, and the supernatant was mixed with Laemmli buffer containing 189 mM of Tris-HCl (pH 6.8), 6% of SDS, 30% of glycerol 0.0045% of bromophenol blue, and 15% of 2-mercaptoethanol and heated at 95 °C for 5 min. The protein concentration of the solution before mixing Laemmli buffer was determined using the Bradford method.

Electrophoresis and transfer were performed using Mini-PROTEIN Tetra System (Bio-Rad, Hercules, CA, USA) and Mini Trans-Blot^®^ Cell (Bio-Rad), respectively. The membrane was blocked with PVDF Blocking Reagent for Can Get Signal^®^ (TOYOBO, Osaka, Japan). The primary and secondary antibodies were diluted with Can Get Signal^®^ Immunoreaction Enhancer Solution (TOYOBO). The antibody-conjugated membrane was developed with ECL Prime Western Blotting Detection Reagent (GE Healthcare, Chicago, IL, USA) for 5 min, and the image was obtained using Ez-Capture MG (ATTO, Tokyo, Japan).

The primary antibodies (all diluted 1:1000) anti-FBXO32 (#ab168372; Abcam, Cambridge, UK), anti-MuRF1 (#sc-398608; Santa Cruz Biotechnology, Dallas, TX, USA), and anti-α tubulin (#3873; Cell Signaling Technology, Danvers, MA, USA) were used. The secondary antibodies anti-rabbit IgG (#NA934) and anti-mouse IgG (#NA931) were purchased from GE healthcare.

### 2.7. LDH Assay

Cell cytotoxicity was measured via the LDH assay (#299-50601; Wako Pure Chemical Industries, Osaka, Japan). After incubation with DEX and TE fraction, the medium was collected into 1.5 mL tubes and cellular LDH was collected with medium containing 1% Tween-20. LDH in the medium and cell was measured according to the manufacturer’s instructions, and the absorbance (Ab) was measured at 560 nm using a spectrophotometer (Thermo Fisher Scientific). Cytotoxicity was calculated using the following formula: Cytotoxicity (%) = (Abs in medium)/(Abs in medium + Abs in cells) × 100.

### 2.8. Statistical Analysis

The results are expressed as the mean ± standard error. In animal experiments, we performed a multiple comparison test using the Tukey–Kramer test. In cell cultivation, the main effects of DEX and TE or TE fraction, and their interaction were determined by a two-way analysis of variance (two-way ANOVA). Where an interaction effect was detected, we performed the Tukey–Kramer test for a multiple comparison. Statistical significance was set at *p* < 0.05.

## 3. Results

### 3.1. Effects of TE on Body Weight and Muscle Weight in DEX-Treated Mice

**Figure 1:**
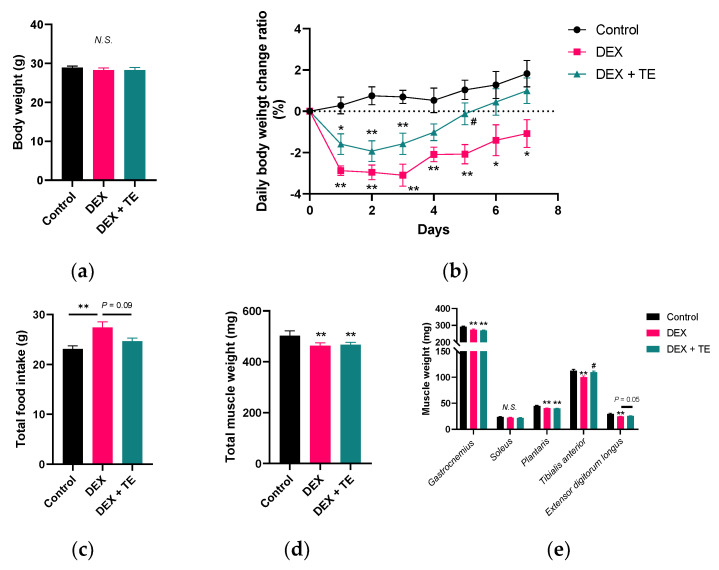
Effects of 2% turmeric extract (TE) supplementation on body weight, food intake, and muscle weight in dexamethasone (DEX)-treated mice. (**a**) Final body weight. (**b**) Daily body weight change ratio. (**c**) Total food intake. (**d**) Total muscle weight. (**e**) Weight of five types of leg muscle. The results are shown as the mean ± standard error and statistical analysis was performed by Tukey–Kramer test. Control group: *n* = 7; DEX and DEX + TE groups: *n* = 6 each. * *p* < 0.05, ** *p* < 0.01 vs. Control, # *p* < 0.05 vs. DEX, N.S.: not significant.

As shown in Figure 1a, DEX injection and TE supplementation did not alter the body weight of the mice. DEX injection significantly decreased daily body weight change ratio for 7 d, and this was slightly restored by TE supplementation, and the value was intermediate between that of the control and DEX groups (*p* < 0.05 on Day 5 and *p* = 0.10 on Day 7 vs. DEX group, Figure 1b). The food intake was significantly increased in DEX group and this change was slightly canceled by TE supplementation (Figure 1c, *p* = 0.09). As shown in Figure 1d, total muscle weight was significantly decreased by DEX injection but was not affected by TE supplementation. DEX injection significantly decreased the weight of the *gastrocnemius*, *plantaris*, *tibialis anterior*, and *extensor digitorum longus* muscles but did not alter that of the *soleus* muscle (Figure 1e). TE supplementation suppressed the muscle weight reduction in the *tibialis anterior* (*p* < 0.05) and *extensor digitorum longus* muscles (*p* = 0.05) but had no effect on the weight of the other three muscles.

### 3.2. DNA Microarray Analysis of DEX-Injected and TE-Supplemented Mice

**Figure 2:**
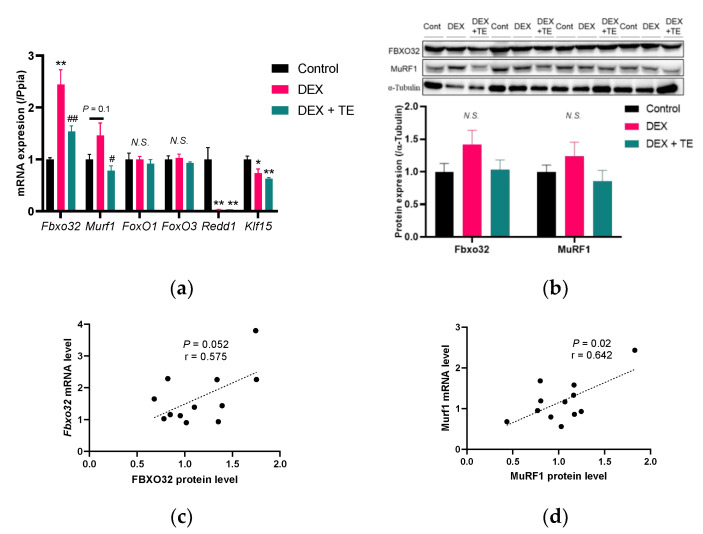
Effects of 2% turmeric extract (TE) supplementation on gene and protein expression of atrophy-related factors in the tibialis anterior muscle of dexamethasone (DEX)-treated mice. (**a**) Gene expression of Fbxo32, Murf1, FoxO1, and FoxO3. (**b**) Protein expression of FBXO32 and MuRF1. (**c**,**d**) are the correlation between mRNA and protein expression of FBXO32 and MuRF1. The results are shown as the mean ± standard error and statistical analysis was performed by Tukey–Kramer test. The correlation test was performed by Pearson correlation test. Control group: *n* = 7; DEX and DEX + TE groups: *n* = 6 each in the PCR experiment and *n* = 4 in Western blotting. * *p* < 0.05, ** *p* < 0.01 vs. Control, # *p* < 0.05, ## *p* < 0.01 vs. DEX, N.S.: not significant.

We carried out DNA microarray analysis in the *tibialis anterior* muscle to understand the functional mechanism underlying the suppressive effects of TE on muscle atrophy in mice. Compared with the control group, DEX injection increased the expression of 264 probes and decreased that of 185 probes, while TE supplementation increased the expression of 40 probes and decreased that of 28 probes (Figure S1). Canonical pathway analysis did not reveal pathways directly related to muscle physiology and metabolism. However, upstream analysis revealed that DEX injection activated the FoxO3 signaling pathway (Table 1), which is important in DEX-induced muscle atrophy [19,20]. Notably, TE supplementation activated the IGF-1 pathway (Table 2), which has a suppressive effect on muscle atrophy [21,22]. In both “FoxO3” and “IGF-1” categories, F-box protein 32 (*Fbxo32*) was listed. Real-time RT-PCR analysis showed that *Fbxo32* expression was increased by DEX injection, and this increase was suppressed by TE supplementation (Figure 2a). Similarly, *Murf1* expression was significantly suppressed by TE supplementation compared with the DEX-treated group. The expression of *FoxO1* and *FoxO3*, which are transcriptional factors of FBXO32 and MurF1, respectively, was not altered by DEX injection and TE supplementation. Furthermore, regulated in development and DNA damage responses 1 (*Redd1*) and Krüppel-like factor 15 (*Klf15*), which have been found to be upregulated by DEX treatment and related to muscle atrophy [7,23,24,25], were significantly decreased by DEX injection but were not altered by TE supplementation. The protein level of FBXO32 and MurF1 showed similar patterns with gene expression, but these were not significant in all experimental groups (Figure 2b). Furthermore, correlation test indicated a strong positive correlation between mRNA and protein level in FBXO32 (r = 0.575, *p* = 0.05, Figure 2c) and MuRF1 (r = 0.642, *p* = 0.02, Figure 2d) expression. These results suggest that TE supplementation suppresses the expression of FBXO32 and MurF1 in DEX-treated mice.

### 3.3. Effects of TE on FBXO32 and MurF1 Expression in DEX-Treated C2C12 Cells

**Figure 3:**
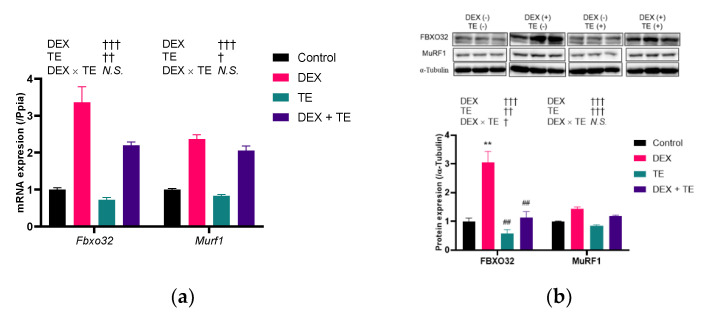
Effects of 200 µM of turmeric extract (TE) on gene and protein expression of atrophy-related factors in dexamethasone (DEX)-treated C2C12 myotubes. (**a**) Gene expression of Fbxo32 and Murf1. (**b**) Protein expression of FBXO32 and MuRF1. The results are shown as the mean ± standard error. PCR experiment: *n* = 4; Western blotting: *n* = 3. Statistical analysis was performed by two-way ANOVA, and where an interaction effect was detected, multiple comparison test was performed by Tukey–Kramer test. The results of two-way ANOVA and multiple comparison test are shown above and in the figure, respectively. † *p* < 0.05, †† *p* < 0.01, ††† *p* < 0.001 in two-way ANOVA, ** *p* < 0.01 vs. Control, ## *p* < 0.01 vs. DEX in multiple comparison test, N.S.: not significant.

We then performed an in vitro study using C2C12 myotubes to investigate the effects of TE on FBXO32 and MurF1 expression. As shown in Figure 3a, the expression of *Fbxo32* and *MurF1* was increased by DEX treatment (*p* < 0.001) but was decreased by TE supplementation (*p* < 0.05). No interactive effects between the expression of the two genes were observed. The protein level of FBXO32 was significantly increased by DEX treatment, and this increase was significantly suppressed by TE supplementation (Figure 3b). Similarly, that of MurF1 was increased by DEX treatment but was decreased by TE supplementation. However, no interaction effects were observed in MurF1 at the protein level.

### 3.4. Effects of Five TE Fractions on FBXO32 and MurF1 Expression in DEX-Treated C2C12 Cells

**Figure 4:**
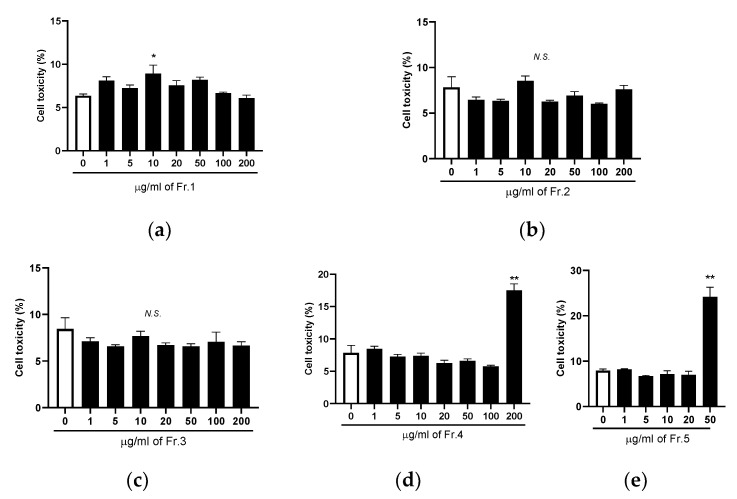
Effects of five turmeric extract (TE) fractions on cell cytotoxicity in C2C12 myotubes. (**a**–**e**) Different concentrations of Fr.1, Fr.2, Fr.3, Fr.4, and Fr.5 were added to C2C12 myotubes for 12 h, after which lactate dehydrogenase assay was performed. The results are shown as the mean ± standard error (*n* = 4), and statistical analysis was performed by Tukey–Kramer test. * *p* < 0.05, ** *p* < 0.01 vs. 0 µg/mL, N.S.: not significant.

To determine the concentration of TE fractions, LDH assay-based cell cytotoxicity was examined. As shown in Figure 4, 10 µg/mL of Fr.1 significantly increased cell cytotoxicity compared with untreated cells, but the effect was not dose dependent. No toxic effect was observed in various concentrations of Fr.2 and Fr.3. The cell cytotoxicity was significantly increased by the addition of 200 µg/mL of Fr.4 and 50 µg/mL of Fr.5. Thus, we chose 5 and 200 µg/mL of Fr.1, 200 µg/mL of Fr.2 and Fr.3, 100 µg/mL of Fr.4, and 20 µg/mL of Fr.5 to evaluate the effect of TE fractions on Fbxo32 and MurF1 expression in DEX-treated C2C12 myotubes.

**Figure 5:**
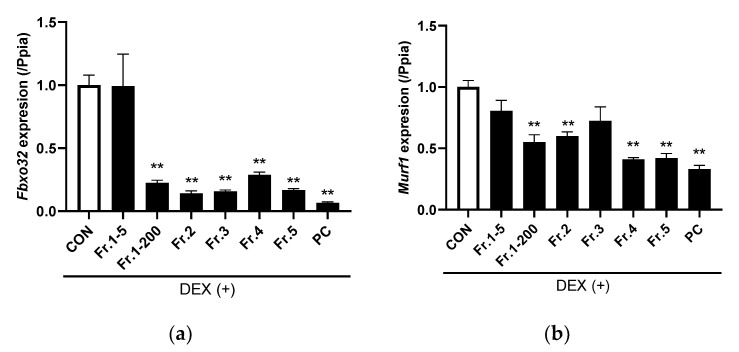
Effects of five turmeric extract (TE) fractions on the gene expression of muscle-specific ubiquitin ligase in dexamethasone (DEX)–treated C2C12 myotubes. (**a**,**b**) Gene expression of Fbxo32 and Murf1, respectively. The positive control group was treated with 10 nM of IGF-1 for 12 h. The results are shown as the mean ± standard error (*n* = 4), and statistical analysis was performed by Tukey-Kramer test. ** *p* < 0.01 vs. Control, N.S.: not significant.

**Figure 6:**
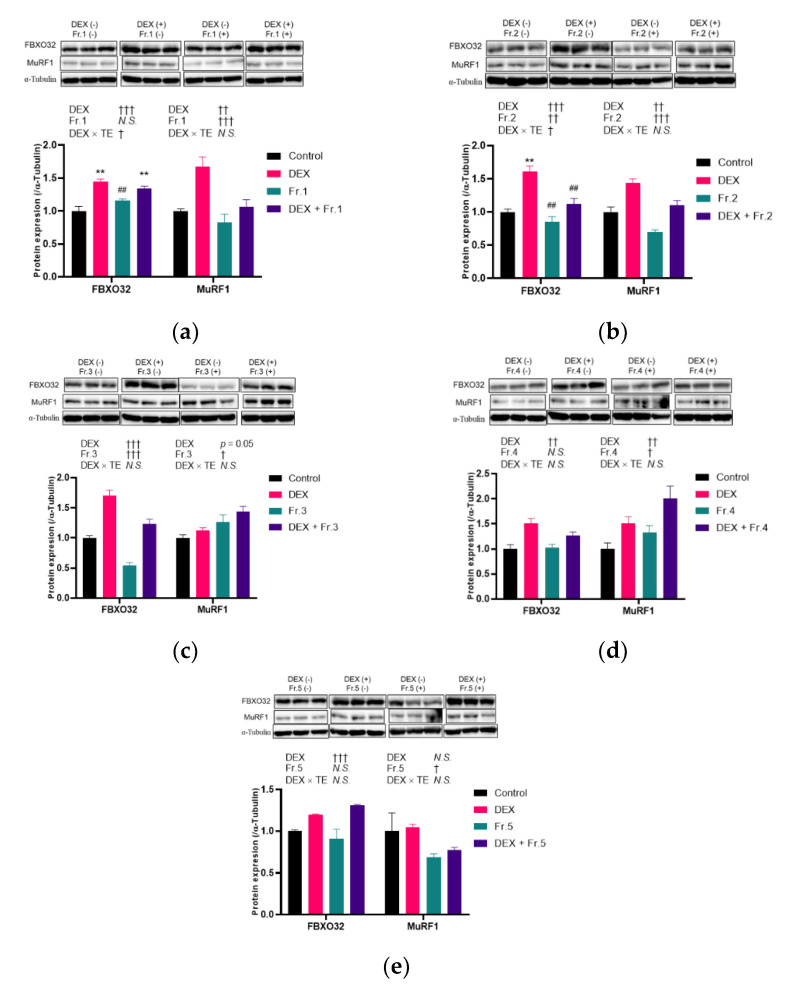
Effects of five turmeric extract (TE) fractions on protein expression of FBXO32 and MuRF1 in dexamethasone (DEX)-treated C2C12 myotubes. The myotubes were incubated with Fr.1 (**a**), Fr.2 (**b**), Fr.3 (**c**), Fr.4 (**d**), and Fr.5 (**e**) for 12 h under DEX co-treatment conditions, and FBXO32 and MuRF1 expressions were investigated. The results are shown as the mean ± standard error, and *n* = 3. Statistical analysis was performed by two-way ANOVA, and where an interaction effect was detected, multiple comparison test was performed by Tukey–Kramer test. The results of two-way and one-way ANOVA are shown below and in the figure, respectively. † *p* < 0.05, †† *p* < 0.01, ††† *p* < 0.001 in two-way ANOVA, ** *p* < 0.01 vs. Control, ## *p* < 0.01 vs. DEX in multiple comparison test, N.S.: not significant.

In DEX-treated cells, the five TE fractions, except for 5 µg/mL of Fr.1, significantly decreased *Fbxo32* and *MurF1* expression (Figure 5a,b). Hence, 5 µg/mL of Fr.1 was not included in subsequent experiments. The FBXO32 protein level was increased by DEX treatment (*p* < 0.001) but was not altered by Fr.1 treatment (Figure 6a). In contrast, the MurF1 protein level was increased by DEX (*p* = 0.001) and decreased by Fr.1 treatment (*p* < 0.001). Fr.2 supplementation decreased the FBXO32 protein level (*p* = 0.002), and an interaction effect between DEX and Fr.2 was observed (*p* = 0.03, Figure 6b). One-way ANOVA revealed that Fr.2 supplementation suppressed the increase in FBXO32 protein level induced by DEX treatment. MurF1 protein level was decreased by Fr.2 supplementation (*p* < 0.001). Meanwhile, Fr.3 supplementation decreased FBXO32 protein level (*p* < 0.001) but increased MurF1 protein level (*p* = 0.01, Figure 6c). Fr.4 and Fr.5 supplementation did not alter FBXO32 protein level but increased MuRF1 protein level (*p* = 0.04 and *p* = 0.03, respectively, Figure 6d,e. Therefore, only Fr.2 has a suppressive effect on both FBXO32 and MuRF1 protein expression.

## 4. Discussion

The present study showed that TE ameliorated DEX-induced muscle atrophy in mice. TE was also found to suppress FBXO32 and MurF1 expression in the skeletal muscle of mice and in C2C12 cells. To date, several studies have shown that turmeric has anti-inflammatory, anti-oxidative stress, and anti-diabetic effects [11]; however, studies on its effect on muscle atrophy are limited. Thus, this study demonstrated the novel function of turmeric in the maintenance of human health.

Notably, muscle atrophy caused by aging occurs in type II fiber [26]. In our data, amelioration of muscle weight reduction by TE supplementation was observed in the *tibialis anterior* and *extensor digitorum longus* muscles in DEX-treated mice. In terms of gene expression, TE supplementation was found to reduce Fbxo32 and/or MurF1 expression in the *tibialis anterior*, *extensor digitorum longus*, and *gastrocnemius* muscles but not in the *soleus* muscle (Figure 2 and Figure S2), which implies that long term supplementation of TE could improve the muscle weight of the *gastrocnemius* muscle, the heaviest muscle in the leg of mice. Previous morphological and enzymatic studies have shown that in C57BL/6J mice, the *soleus* muscle has type IIA and type I muscle fibers, while *tibialis anterior*, *extensor digitorum longus*, and *gastrocnemius* muscles have predominantly type IIB and type IIDB fibers [27]. Therefore, TE has a suppressive effect on muscle atrophy in type II muscle fibers and may be an effective nutritional strategy against muscle atrophy caused by aging.

Similar effects on FBXO32 and MurF1 expression were observed in mice experiment and cell cultivation test using Fr.2, which suggests that a component of Fr.2 is absorbed by the gastrointestinal tract and regulates these expressions. Curcumin is an important curcuminoid in turmeric, and its supplementation suppressed muscle atrophy in streptozotocin-induced type 1 diabetes mellitus mice [16]. Bisacurone, also a bioactive compound in turmeric, improved hepatic lipid metabolism and inflammatory diseases [14,15]. However, since TE contains less than 0.1% curcuminoid, curcumin may not be an effective compound to suppress muscle atrophy in our study. Furthermore, we carried out liquid chromatography- and gas chromatography-mass spectrometry analyses to determine the bioactive compound in Fr.2, which had the most effect on FBXO32 and MurF1 expression. In both analyses, curcumin and bisacurone were not detected, which suggests that Fr.2 contains a novel bioactive compound in TE and/or turmeric that suppresses muscle atrophy. Metabolomic analysis was performed to identify the novel bioactive compound, and five candidates, namely ρ-cymene, ethanol, 1-(2,5-dimethylphenyl)-, benzene, 1-methyl-4-(1-methylethenyl)-, benzeneacetic acid, methyl ester, and thymol methyl ether, were found. To date, no studies have reported the effect of these five compounds on muscle atrophy. However, ρ-cymene and thymol are known to have suppressive effects against several physiological disorders, such as oxidative stress and inflammation [28,29], which are closely linked to sarcopenia [30]. Given that these two molecules are present in the essential oil of C. longa [31,32], they may be responsible for the suppressive effects of TE on muscle weight loss and FBXO32 and MuRF1 expression. Nevertheless, further studies are required to identify the bioactive compound in turmeric that improves muscle atrophy.

In contrast, the other four TE fractions did not improve the protein expression of both FBXO32 and MuRF1, which is inconsistent with the mRNA results. A previous study had indicated that the mRNA levels can only explain 40–70% of the protein abundance [33]. These results suggested that these four fractions might contain compounds affecting intracellular amino acid concentration, protein translation, or protein halftime. Furthermore, Fr.3 suppressed FBXO32 expression, and Fr.1 and Fr.5 improved MuRF1 expression in DEX-treated C2C12 myotubes, which suggests the presence of other effective molecules in these TE fractions. Overall, TE possibly contains several bioactive compounds that can regulate muscle physiology and metabolism.

DEX induces FBXO32 and MuRF1 expression through the glucocorticoid receptor (GR)-FoxO signaling pathway [5,10]. In DEX-treated mice, the transcription levels of *FoxOs*, *Klf15*, and *Redd1* were not altered by TE supplementation. Furthermore, the protein level of GR was decreased by DEX treatment, but no change was observed in TE-supplemented mice compared with DEX-treated mice. Meanwhile, the phosphorylation ratio of protein kinase B and mammalian target of rapamycin was not altered by DEX injection or TE supplementation. A possible mechanism is that TE affects the protein level of peroxisome proliferator-activated receptor-γ coactivator 1-α, which inhibits FoxO3 activity [10]. Further biological experiments should be conducted to elucidate the effect of TE in skeletal muscle.

## 5. Conclusions

The present study indicates that TE ameliorates DEX-induced muscle atrophy in vivo and in vitro. The findings show the novel function of turmeric, which may be applied in the development of nutritional strategies to improve and/or delay aging-associated muscle atrophy.

## Figures and Tables

**Table 1:** Gene list of “FoxO3”.

Gene Symbol	Gene Name	Log Ratio (Dex vs. Con)
*LCN2*	Lipocalin 2	7.43
*CDKN1A*	Cyclin Dependent Kinase Inhibitor 1A	2.134
*FOS*	Fos Proto-Oncogene, AP-1 Transcription Factor Subunit	1.912
*GADD45B*	Growth Arrest And DNA Damage Inducible Beta	1.682
*Mt1*	Metallothionein-1	1.535
*Mt2*	Metallothionein-2	1.291
*TPX2*	TPX2 Microtubule Nucleation Factor	1.242
*IRS2*	Insulin Receptor Substrate 2	1.079
*FASN*	Fatty Acid Synthase	1.076
*PPP1R15A*	Protein Phosphatase 1 Regulatory Subunit 15A	1.049
*EGR1*	Early Growth Response 1	0.982
*UBC*	Ubiquitin C	0.974
*FBXO32*	F-Box Protein 32	0.906
*MTHFD2*	Methylenetetrahydrofolate Dehydrogenase (NADP+ Dependent) 2	0.902
*GABRR2*	Gamma-Aminobutyric Acid Type A Receptor Subunit Rho2	0.837
*EIF4EBP1*	Eukaryotic Translation Initiation Factor 4E Binding Protein 1	0.79
*JUNB*	JunB Proto-Oncogene, AP-1 Transcription Factor Subunit	0.738
*BCL2L11*	BCL2-Like 11	0.733
*OPTN*	Optineurin	0.677
*CDKN1B*	Cyclin Dependent Kinase Inhibitor 1B	0.668
*MAP1LC3A*	Microtubule Associated Protein 1 Light Chain 3 Alpha	0.633
*PAK1*	P21 (RAC1) Activated Kinase 1	−0.664
*PTEN*	Phosphatase And Tensin Homolog	−0.676
*CCNE2*	Cyclin E2	−0.726
*GRB14*	Growth Factor Receptor Bound Protein 14	−0.772
*VCAM1*	Vascular Cell Adhesion Molecule 1	−0.784
*FMOD*	Fibromodulin	−0.799
*IGF1*	Insulin-Like Growth Factor 1	−0.799
*CCND1*	Cyclin D1	−0.816
*FN1*	Fibronectin 1	−1.075
*NFKBIA*	NFKB Inhibitor Alpha	−1.296
*TCIM*	Transcriptional And Immune Response Regulator	−1.31
*DDIT4*	DNA Damage Inducible Transcript 4	−4.14

In upstream analysis using IPA software, the “FoxO3” category was listed in the comparison between the control and DEX groups. The activation Z score of this category was 2.795.

**Table 2:** Gene list of “IGF1”.

Gene Symbol	Gene Name	Log Ratio (DEX + TE vs. DEX)
*SLC25A25*	Solute Carrier Family 25 Member 25	1.16
*ADIPOQ*	Adiponectin, C1Q And Collagen Domain Containing	1.041
*SCD*	Stearoyl-CoA Desaturase	0.888
*TGFBI*	Transforming Growth Factor Beta Induced	0.787
*IGF1*	Insulin-Like Growth Factor 1	0.733
*C4A/C4B*	Complement C4B (Chido Blood Group)	0.679
*COL3A1*	Collagen Type III Alpha 1 Chain	0.677
*FBXO32*	F-Box Protein 32	−0.846
*SLC20A1*	Solute Carrier Family 20 Member 1	−0.997

In upstream analysis using IPA software, the “IGF1” category was listed in the comparison between the DEX and DEX + TE groups. The activation Z score of this category was 1.991.

## Data Availability

The datasets presented in this study can be found in the NCBI’s Gene Expression Omnibus (GEO) online repositories under accession number GSE209528.

## Supplementary Materials

The following supporting information can be downloaded at: https://www.mdpi.com/article/10.3390/nu14193979/s1, Figure S1: Venn diagram of detected probes in DNA microarray analysis; Figure S2: Effects of 2% TE supplementation on gene expression of Fbxo32 and Murf1 in *Gastrocnemius* (a), *Extensor digitorum longus* (b), *Soleus* (c), and *Plantaris* muscle (d) of DEX-challenged mice; Table S1: Diet composition; Table S2: Primer sequences.
