# Supplementary material for: Suppressive Effects of Turmeric Extract on Muscle Atrophy in Dexamethasone-Treated Mice and Myotubes
Source: Nutrients. 2022 Sep 25;14(19):3979. doi: 10.3390/nu14193979 (PMC9571062; doi:10.3390/nu14193979)
Supplement: Supplementary file 1 [file nutrients-14-03979-s001.zip › nutrients-1915692-supplementary.pdf]

Supplementary Materials

1. Supplementary Figure

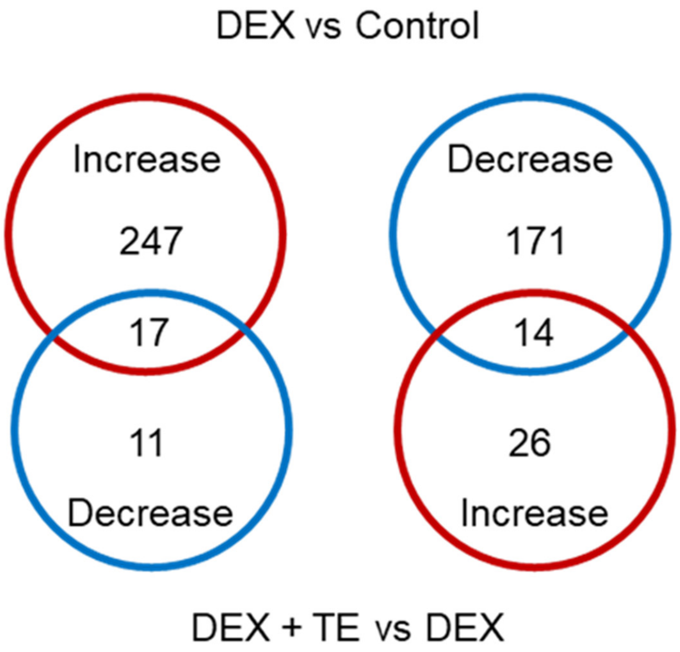

Figure S1. Venn diagram of detected probes in DNA microarray analysis.

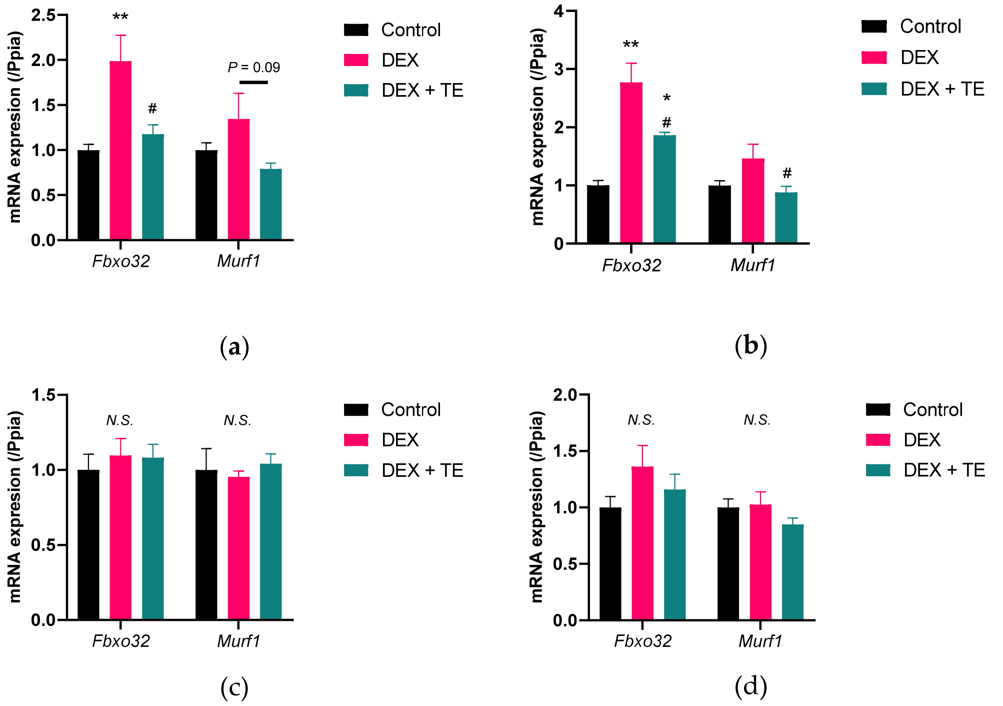

Figure S2. Effects of 2 % TE supplementation on gene expression of *Fbxo32* and *Murf1* in *Gastrocnemius* (a), *Extensor digitorum longus* (b), *Soleus* (c), and *Plantaris* muscle (d) of DEX-challenged mice. The results are shown as the mean ± standard error and statistical analysis was performed by Tukey-Kramer test. *$p < 0.05$, **$p < 0.01$ vs Control, $^{\#}p < 0.05$ vs DEX, N.S.: not significant.

2. Supplementary Tables

Table S1. Diet composition

Component (%)	Control diet	2% TE supplemented diet
Casein	20.0	20.0
β-corn starch	39.75	37.75
α-corn starch	13.2	13.2
Soybean oil	7.0	7.0
Sucrose	10.25	10.25
Cellulose	5.0	5.0
Vitamin mixture*	1.0	1.0
Mineral mixture*	3.5	3.5
L-cystine	0.3	0.3
TE powder	0.0	2.0

* AIN-93 prescription (Oriental Yeast Co., Ltd., Tokyo, Japan). TE: Turmeric extract.

Table S2. Primer sequences

Gene		Primer sequence (5′-3′)
For mice		
Fbxo32	*Sense*	GCTGGTGGGCAACATTAACA
	Antisense	GTTGTAAGCACACAGGCAGGTC
Murf1	*Sense*	CTGAGTAACTGCATCTCCATGCT
	Antisense	TCCTTCACCTGGTGGCTATTC
FoxO1	*Sense*	ACATTTCGTCCTCGAACCAGCTCA
	Antisense	ATTTCAGACAGACTGGGCAGCGTA
FoxO3	*Sense*	TCGTCTCTGAACTCCTTGCGT
	Antisense	TGGAGTGTCTGGTTGCCGT
Redd1	*Sense*	CCAGACAAGAGGGCCTTGA
	Antisense	CCATCCAGGTATGAGGAGTCTT
Klf15	*Sense*	CCAAGAGCAGCCACCTCCAA
	Antisense	CAACTCATCTGAGCGGGAAA
Ppia	*Sense*	CAAATGCTGGACCAAACACAA
	Antisense	TCACCTTCCCAAAGACCACA

Fbxo32: F-Box Protein 32, Murf1: Muscle RING-finger protein-1, Foxo: Forkhead box O, Redd1: regulated in development and DNA damage responses 1, Klf15: Krüppel-like factor 15, Ppia: Peptidyprolyl isomerase A
